# Impact of Care Initiation Model on Emergency Department Orders and Operational Metrics: Cohort Study

**DOI:** 10.5811/westjem.59340

**Published:** 2023-07-12

**Authors:** Andy Hung-Yi Lee, Rebecca E. Cash, Alice Bukhman, Dana Im, Damarcus Baymon, Leon D. Sanchez, Paul C. Chen

**Affiliations:** * Brigham and Women’s Hospital, Department of Emergency Medicine, Boston, Massachusetts, USA; † Harvard Medical School, Boston, Massachusetts, USA; ‡ Massachusetts General Hospital, Department of Emergency Medicine, Boston, Massachusetts, USA; § Brigham and Women’s Faulkner’s Hospital, Department of Emergency Medicine, Jamaica Plain, Massachusetts, USA

## Abstract

**Introduction:**

Emergency departments (ED) employ many strategies to address crowding and prolonged wait times. They include front-end Care Initiation and clinician-in-triage models that start the diagnostic and therapeutic process while the patient waits for a care space in the ED. The objective of this study was to quantify the impact of a Care Initiation model on resource utilization and operational metrics in the ED.

**Methods:**

We performed a retrospective analysis of ED visits at our institution during October 2021. Baseline characteristics were compared with Chi-square and quantile regression. We used *t*-tests to calculate unadjusted difference in outcome measures, including number of laboratory tests ordered and average time patients spent in the waiting room and the ED treatment room, and the time from arrival until ED disposition. We performed propensity score analysis using matching and inverse probability weighting to quantify the direct impact of Care Initiation on outcome measures.

**Results:**

There were 2,407 ED patient encounters, 1,191 (49%) of whom arrived during the hours when Care Initiation was active. A total of 811 (68%) of these patients underwent Care Initiation, while the remainder proceeded directly to the main treatment area. Patients were more likely to undergo Care Initiation if they had lower acuity and lower risk of admission, and if the ED was busier as measured by the number of recent arrivals and percentage of occupied ED beds. After adjusting for patient-specific and department-level covariates, Care Initiation did not increase the number of diagnostic laboratory tests ordered. Care Initiation was associated with increased waiting room time by 1.8 hours and longer time from arrival until disposition by 1.3 hours, but with decreased time in the main treatment area by 0.6 hours, which represents a 15% reduction.

**Conclusion:**

Care Initiation was associated with a 15% reduction in time spent in the main ED treatment area but longer waiting room time and longer time until ED disposition without significantly increasing the number of laboratory studies ordered. While previous studies produced similar results with Care Initiation models accessing all diagnostic modalities including imaging, our study demonstrates that a more limited Care Initiation model can still result in operational benefits for EDs.

## INTRODUCTION

Emergency departments (ED) often experience significant crowding, which can lead to prolonged patient wait times and worse outcomes.^1^,^2^ This has been exacerbated by increasing numbers of admitted patients boarding in the ED while waiting for an inpatient bed. These inpatient boarders consume ED resources including beds and staff and slow the department’s ability to manage newly arriving patients.^3^,^4^ Multiple strategies have been employed by EDs across the country to prevent delays in care, including the use of Care Initiation and clinician-in-triage models.^5^,^6^

Care Initiation and clinician-in-triage models involve placing an emergency physician or physician assistant (PA) at the point of triage who can rapidly screen patients and determine initial plans of care.^7^ The clinician is often empowered to treat and directly discharge patients with straightforward complaints and initiate evaluation and treatment for patients with more complex complaints.^8^ Multiple studies in the literature describe single-site implementation of these models in different ED settings,^9^–^11^ but their impact may depend on capabilities and resources dedicated to these front-end models and may also depend on specific characteristics of the individual sites, such as patient populations, staffing levels, and bottlenecks in care.^7^ Moreover, their primary focus has been on operational metrics such as left without being seen (LWBS) rates, door-to-clinician time, and ED length of stay,^12^ and few of these studies attempted to quantify the effect of Care Initiation models on utilization of ED resources.

The Care Initiation system at our institution differs from many others in that it is restricted to laboratory diagnostic testing and is deployed at an academic medical center with a highly complex transplant and oncologic population. Our objective in this study was to assess the impact of Care Initiation at our institution by comparing the number of diagnostic tests ordered and to assess whether this strategy offers the same operational improvements seen in other studies.

## METHODS

### Study Design and Setting

We performed a retrospective cohort study of patients presenting to our academic ED in the Northeastern United States in the month of October 2021. The ED is a Level I trauma center, comprehensive stroke center; it is affiliated with a comprehensive oncologic treatment center and had an annual census of ≈65,000 ED visits in 2021. The study was approved by the institutional review board (Protocol 2022P000264) and performed according to STROBE guidelines.^13^

**Figure 1:**
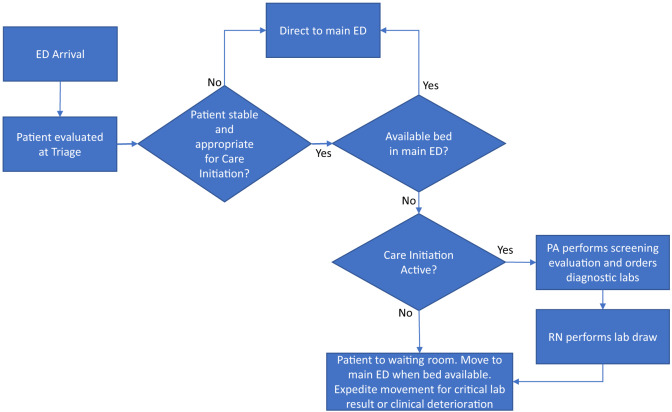
Care Initiation model.

The Care Initiation program in the ED was implemented in January 2021 and was designed to start laboratory work-up including blood and urine studies on waiting room patients. The program operated from noon to midnight on weekdays and was staffed by a PA with at least two years of experience working in the ED. The PA would screen all triaged patients in the waiting room and based on their clinical evaluation would order lab studies based on what they determined would be needed as part of the ED work-up. The required blood and urine samples would be obtained and the patient returned to the waiting room, where the patient continued to wait for a care space in the ED (Figure 1). The Care Initiation PA was not authorized to order radiologic studies in this program. When Care Initiation was not active, patients would wait in the waiting room until an open care space was available in the ED as per usual care.

**Population Health Research Capsule**

What do we already know about this issue?

*Many emergency departments (ED) have implemented Care Initiation to start the diagnostic work-up of patients while they are still in the waiting room*.

What was the research question?

*We analyzed how Care Initiation impacts the number of lab orders and ED operational metrics*.

What was the major finding of the study?

*Care Initiation did not increase lab order numbers and was associated with 0.6 fewer hours in an ED bed*.

How does this improve population health?

*Care Initiation can help address ED capacity constraints without significantly increasing the cost of care*.

### Outcome Measures and Data Collection

We obtained data were obtained from the electronic health record (Epic Systems Corporation, Verona, WI) for all ED encounters at our institution in October 2021. The ED encounters were categorized based on whether Care Initiation was active and whether the encounter proceeded through the Care Initiation program. We excluded ED encounters in which patients arrived via emergency medical services (ground or air) or no lab work was ordered as they were not considered for Care Initiation at triage. Individual patient variables included patient age, gender, race/ethnicity, and Emergency Severity Index (ESI). Operation-level variables included number of ED arrivals in the prior four hours at time of patient presentation and percentage of ED bed occupancy at time of ED presentation. Timestamps were obtained for each patient encounter, which included ED arrival, ED rooming, and ED disposition. Primary outcome variables included the number of diagnostic lab tests ordered prior to ED disposition and the length of time the patient spent in the main ED. The lab tests included were compiled from a list of the 25 most frequently ordered lab tests in the ED at our institution (Supplement 1). Secondary outcome variables included ED waiting time and time from ED arrival to ED disposition.

### Data and Statistical Analysis

We analyzed data at the encounter level and performed statistical analyses in Stata 15 (StataCorp, College Station, TX). Comparisons between continuous variables were calculated for demographic variables using medians and quantile regressions and for outcome variables using Student *t*-tests. We calculated comparisons between categorical variables using chi-square tests, and we used logistic regression to calculate the unadjusted and adjusted treatment effect of Care Initiation. Because of the observational nature of the data and unequal probability of selection for Care Initiation, which can introduce bias, we performed several sensitivity analyses. Propensity score matching was performed based on patient age, gender, ESI, number of ED arrivals within the prior four hours, and percentage occupancy of ED beds as covariates in a logistic model to quantify the matched effect of Care Initiation on outcome measures. Additional sensitivity analysis of the outcome measures was performed using inverse probability weighting.

## RESULTS

Excluding patients who arrived via EMS, there were 2,407 ED patient encounters at our institution where lab testing was ordered (Table 1). Of those 2,407 encounters, 1,191 occurred when the Care Initiation program was active, and 811 (68%) underwent Care Initiation while the other 380 encounters (32%) proceeded directly to the ED. Among patients who arrived during Care Initiation hours, patients who underwent Care Initiation were more likely to be female (65% vs 54%), had lower acuity by ESI (median 3 vs 2), and were less likely to be admitted to the hospital (37% vs 45%) (*P* < 0.05 for all comparisons). Patients were also more likely to bypass Care Initiation when there were fewer ED arrivals in the prior four hours (medians 61 vs 64) and when the percentage of occupied ED beds was lower (median 85% vs 86%) (*P* < 0.05 for all comparisons). Moreover, patients who arrived during Care Initiation hours were more likely to be White than Black and tended to have higher acuity by ESI and rate of admission compared to patients who arrived outside Care Initiation hours. Outside Care Initiation hours, there also tended to be fewer arrivals in the prior four hours and a smaller percentage of occupied ED beds.

Patients who proceeded through Care Initiation had fewer lab tests ordered prior to ED disposition than patients who proceeded directly to the main treatment area (6.8 vs 7.3 lab orders, *P* < 0.01). This difference was not statistically significant after accounting for patient-specific variables of age, gender, and ESI acuity (Table 2). Patients who arrived during Care Initiation hours had more lab tests ordered than those who arrived outside of Care Initiation hours (7.0 vs 6.5, *P* < 0.01).

Patients who experienced Care Initiation had a significantly longer time in the waiting room (3.2 vs 1.2 hours, *P* < 0.01) and longer time from ED arrival to ED disposition (6.7 vs 5.1 hours, *P* < 0.01) compared to patients who did not proceed through Care Initiation (Table 3). These differences decreased in magnitude but persisted even after adjusting for patient-specific variables of age, gender, and ESI acuity. However, they had a shorter length of time spent in the main ED treatment area (3.6 vs 3.9 hours, *P* < 0.01), and this persisted after accounting for patient-specific variables of age, gender, and ESI acuity. During hours when Care Initiation was not active, patients experienced shorter length of time in the waiting room (1.2 vs 2.5 hours, *P* < 0.01) and less time from arrival to ED disposition (5.4 vs 6.2 hours) but longer time in the main treatment area (4.2 vs 3.7 hours, p < 0.01).

Given intrinsic differences between patient groups based on time of arrival and whether they proceed through Care Initiation, we performed propensity score analysis with matching and inverse probability weighting to evaluate the direct impact of Care Initiation on outcome measures. After matching patients based on individual patient factors of age, gender, and ESI and based on ED-level operational variables of number of ED arrivals in prior four hours and percentage occupancy of ED beds, we found that Care Initiation did not significantly affect the number of lab studies ordered. However, Care Initiation did significantly impact time metrics for patients by on average shortening time in the main treatment area for patients by 0.6 hours (*P* < 0.01) and lengthening time in the waiting room by 1.8 hours (*P* < 0.01) and time from arrival to ED disposition by 1.3 hours (*P* < 0.01). This overall shortened the length of time that patients occupied a bed in the ED by 15%. Covariate balance was acceptable based on standardized differences and variance ratio (Supplement 2). Repeating this analysis using inverse probability weighting produced similar results (Table 3).

## DISCUSSION

Emergency departments considering the implementation of Care Initiation and clinician in triage often have concerns that these processes will increase the overall number of lab tests ordered for a patient. This concern is understandable, as emergency clinicians try to anticipate the likely diagnostic work-up after performing only a brief assessment of the patient. In our study, Care Initiation did not significantly increase the number of lab orders before ED disposition, even after propensity score matching of patient cohorts. We found no broad consensus in previous literature on this topic. When Care Initiation is applied to a broad population, some studies suggest that it is associated with more diagnostic testing including radiology imaging.^11^,^14^ Other studies focusing specifically on patients with abdominal pain have found either no difference or higher use of diagnostic imaging.^15^,^16^ There may also be substantial differences depending on the type of clinician (emergency physician vs advanced practice practitioner [APP] vs triage nurse) performing this Care Initiation role. Two studies found that triage nurses order more tests than physicians,^17^,^18^ while another showed no differences in total number of tests between APPs compared to physicians.^19^ Our study, although limited to a front-end model that involves a PA ordering only lab tests, did not show an increased utilization of laboratory diagnostic testing for patients who proceed through Care Initiation even after accounting for patient-specific factors through propensity score matching. Our results support the role of APPs in Care Initiation and provide reassurance against overutilization of diagnostic resources, although further monitoring particularly with regard to radiology is warranted based on the literature.

We were able to show that Care Initiation shortened the length of time patients spend in an ED bed by 0.6 hours, which is significant given this represents a 15% reduction. However, this came at the cost of increasing a patient’s total time in the ED until disposition, mostly due to longer time in the waiting room. This is consistent with previous literature that showed shorter length of time in an ED bed but a longer time until disposition^20^ when clinicians in triage initiate ED work-up. This is desirable for most EDs as they are able to use time in the waiting room to advance care while minimizing time in an ED bed, which is a scarcer resource. Furthermore, it is significant that while most previous studies observed this reduction of time in an ED bed when the triage clinician had full access to all lab and imaging tests including computed tomography, we were able to demonstrate a similar effect through lab orders alone.

This is notable because waiting for diagnostic imaging resources is often an operational bottleneck that is part of the “critical path” for many patients,^7^ and there may also be logistic challenges for patients in the waiting room to join the queue for diagnostic imaging. These advantages of Care Initiation are in addition to improving other common ED metrics such as reducing door-to-clinician time and LWBS rates, which have been extensively studied in the literature.^21^–^23^

Care Initiation may also have additional benefits for patients who do not go through the process itself. Our data suggests that even the patient cohort who bypassed Care Initiation experienced shorter time in an ED bed before disposition compared to when Care Initiation was not active. This may be because faster throughput of Care Initiation patients through ED beds allows resources to be focused on higher acuity patients. Moreover, having a clinician in triage may allow more frequent reassessment of waiting room patients and identification of those who may decompensate after spending several hours waiting with relatively infrequent monitoring.

## LIMITATIONS

There are several limitations to our study. First, this was a single-center, retrospective cohort study that examined ED encounters occurring over the span of a month and used an institutional-specific form of Care Initiation. Our data, therefore, may not be applicable to all EDs despite sharing many similarities with other models. We have since transitioned toward a Care Initiation model designed to discharge patients directly from the Care Initiation area, which will be the subject of further study. We were also limited in the scope of our data to after deployment of Care Initiation; additional analyses could be performed when comparing pre- and post-implementation outcomes. Moreover, propensity score matching can introduce additional bias into the dataset, but this is most likely ameliorated by reproducing the same results with inverse probability weighting methodology. Finally, our results are likely not applicable to very high-acuity or low-acuity patients due to their deliberate exclusion from Care Initiation models, which is a common practice in EDs across the country.

## CONCLUSION

Overall, Care Initiation at our institution did not significantly increase the number of lab diagnostic tests ordered and resulted in shortening the average length of time spent in an ED bed by 0.6 hours, which is 15% of the average time to ED disposition. Although every ED faces unique challenges in throughput and efficiency, implementation of Care Initiation and other clinician-in-triage programs may offer some relief especially in EDs with prolonged wait times and significant crowding.

## Supplementary Information





## Figures and Tables

**Table 1:** Patient demographics.

	Patients arriving during Care Initiation hours		Patients arriving outside Care Initiation hours
	
	Patients proceeding through Care Initiation	Patients proceeding directly to main ED	All patients
Number of patients (N)	811	380	1,216
Age: median (IQR)	49 (32, 66)	53 (34, 69)	51 (33, 66)
Gender: N (%)			
Female	530 (65)*	206 (54)*	746 (61)
Male	281 (35)*	174 (46)*	470 (39)
Race: N (%)			
Asian	34 (4)	12 (3)	48 (4)
Black	138 (17)	63 (17)	280 (23)
White	473 (58)	238 (63)	646 (53)
Other/multiple	166 (20)	67 (18)	242 (20)
Ethnicity: N (%)			
Hispanic	157 (19)	75 (20)	256 (21)
Non-Hispanic	654 (81)	305 (80)	960 (79)
ESI Acuity: N (%)			
1	0 (0)*	4 (1)*	0 (0)
2	303 (37)*	233 (61)*	509 (42)
3	496 (61)*	128 (34)*	660 (54)
4	12 (1)*	14 (4)*	45 (4)
5	0 (0)*	1 (0)*	0 (0)
Number of ED arrivals in the prior 4 hours: median (IQR)	64 (59, 71)*	61 (52, 69)*	38 (24, 53)
Percentage ED bed occupancy at ED arrival: median percentage (IQR)	86 (83, 89)*	85 (81, 89)*	69 (59, 76)
ED disposition: N (%)			
Admission	301 (37)*	170 (45)*	384 (32)
ED observation	5 (1)*	12 (3)*	23 (2)
Discharge	505 (62)*	198 (52)*	809 (67)

* *P* < 0.05.

*ED*, emergency department; *IQR*, interquartile range; *ESI*, Emergency Severity Index.

**Table 2:** Study outcome measures without cohort matching.

	Patients arriving during Care Initiation hours		Patients arriving outside Care Initiation hours	Care Initiation unadjusted average treatment effect: unadjusted ATE (95% CI)	Care Initiation adjusted average treatment effect: adjusted ATE (95% CI)
	
	Patients proceeding through Care Initiation: average (SD)	Patients proceeding directly to main ED: average (SD)	All patients: average (SD)		
Number of patients (N)	811	380	1216	1191	1182
Number of lab orders prior to ED disposition	6.8 (2.8)^*^	7.3 (3.8)^*^	6.5 (3.2)	−0.6 (−1.0 to −0.1)	−0.3 (−0.8 to 0.1)
Waiting room time in hours	3.2 (1.6)^*^	1.2 (1.6)^*^	1.2 (1.4)	2.0 (1.8 to 2.2)	1.7 (1.5 to 1.9)
Time in main ED in hours	3.6 (2.0)^*^	3.9 (2.1)^*^	4.2 (2.1)	−0.4 (−0.6 to −0.1)	−0.5 (−0.8 to 0.2)
Total time from arrival to disposition in hours	6.7 (2.6)^*^	5.1 (2.6)^*^	5.5 (2.5)	1.6 (1.0 to 1.9)	1.2 (0.8 to 1.5)

** p < 0.05.

*ED*, emergency department; *ATE*, average treatment effect; *CI*, confidence interval.

**Table 3:** Propensity score and inverse probability weighting analysis.

	Propensity score matching		Inverse probability weighting	
	
	Average treatment effect of Care Initiation (95% CI)	*P*-value	Average treatment effect of Care Initiation (95% CI)	*P*-value
Number of lab orders prior to ED disposition: average (SD)	−0.2 (−0.7 to 0.3)	0.39	−0.4 (−0.9 to 0.1)	0.11
Waiting room time in hours: average (SD)	1.8 (1.3 to 2.0)	<0.01	1.6 (1.4 to 1.9)	<0.01
Time in main ED in hours: average (SD)	−0.6 (−0.9 to −0.3)	<0.01	−0.5 (−0.8 to −0.2)	<0.01
Total time from arrival to disposition in hours: average (SD)	1.3 (0.9 to 1.6)	<0.01	1.1 (0.8 to 1.5)	<0.01

*ED*, emergency department; *CI*, confidence interval.
