# Supplementary material for: Impact of Care Initiation Model on Emergency Department Orders and Operational Metrics: Cohort Study
Source: West J Emerg Med. 2023 Jul 12;24(4):703–9. doi: 10.5811/westjem.59340 (PMC10393454; doi:10.5811/westjem.59340)
Supplement: Supplementary file 1 [file wjem-24-703-s001.docx]

Appendix 1: List of lab orders counted for this study

-CBC and differential

-BMP

-Troponin

-NT-proBNP

-LFTs

-Point of care glucose

-UA with reflex culture

-Blood Culture

-Lactate

-Type and Screen

-Urine HCG

-Quantitative HCG, blood

-Venous blood gas (VBG or VBG plus)

-Lipase

-Magnesium

-PT-INR

-PTT

-D-dimer

-Ethanol, blood level

-Toxicology screen, urine

-Procalcitonin

-ESR

-CRP

-TSH with reflex T4

-Phosphorus
