# Supplementary material for: Impact of Care Initiation Model on Emergency Department Orders and Operational Metrics: Cohort Study
Source: West J Emerg Med. 2023 Jul 12;24(4):703–9. doi: 10.5811/westjem.59340 (PMC10393454; doi:10.5811/westjem.59340)
Supplement: Supplementary file 2 [file wjem-24-703-s002.docx]

Appendix 2: Propensity Matching Covariate Balance

|  | Standardized differences | | Variance ratio | |
| --- | --- | --- | --- | --- |
|  | Raw | Matched | Raw | Matched |
| Age | -0.109 | -0.103 | 0.960 | 0.991 |
| Male Sex | -0.225 | -0.024 | 0.911 | 0.988 |
| ESI |  |  |  |  |
| 3 | 0.561 | 0.056 | 1.057 | 0.997 |
| 4 | -0.142 | 0.053 | 0.404 | 1.331 |
| ED Arrivals in Last 4 Hours | 0.454 | -0.051 | 0.551 | 0.785 |
| ED Occupancy (%) | 0.187 | -0.055 | 0.690 | 0.791 |
